# Age of smile: a cross-cultural replication report of Ganel and Goodale (2018)

**DOI:** 10.1007/s41809-020-00072-3

**Published:** 2021-01-10

**Authors:** Naoto Yoshimura, Koichi Morimoto, Mariko Murai, Yusaku Kihara, Fernando Marmolejo-Ramos, Veit Kubik, Yuki Yamada

**Affiliations:** 1 Graduate School of Human-Environment Studies, Kyushu University, 744 Motooka, Nishi ku, Fukuoka, 819-0395 Japan; 2 Japan Society for the Promotion of Science, Tokyo, Japan; 3 Center for Change and Complexity in Learning, The University of South Australia, Adelaide, Australia; 4 Department of Psychology, Bielefeld University, Bielefeld, Germany; 5 Department of Psychology, Stockholm University, Stockholm, Sweden; 6 Faculty of Arts and Science, Kyushu University, Fukuoka, Japan

**Keywords:** Facial expression, Age evaluation, Replication study, Cross-cultural design

## Abstract

Smiling is believed to make people look younger. Ganel and Goodale (Psychon Bull Rev 25(6):612–616, 10.3758/s13423-017-1306-8, 2018) proposed that this belief is a misconception rooted in popular media, based on their findings that people actually perceive smiling faces as older. However, they did not clarify whether this misconception can be generalized across cultures. We tested the cross-cultural validity of Ganel and Goodale’s findings by collecting data from Japanese and Swedish participants. Specifically, we aimed to replicate Ganel and Goodale’s study using segregated sets of Japanese and Swedish facial stimuli, and including Japanese and Swedish participants in groups asked to estimate the age of either Japanese or Swedish faces (two groups of participants × two groups of stimuli; four groups total). Our multiverse analytical approach consistently showed that the participants evaluated smiling faces as older in direct evaluations, regardless of the facial stimuli culture or their nationality, although they believed that smiling makes people look younger. Further, we hypothesized that the effect of wrinkles around the eyes on the estimation of age would vary with the stimulus culture, based on previous studies. However, we found no differences in age estimates by stimulus culture in the present study. Our results showed that we successfully replicated Ganel and Goodale (2018) in a cross-cultural context. Our study thus clarified that the belief that smiling makes people look younger is a common cultural misconception.

## Introduction

The visual system has optimized for facial processing. In particular, facial expressions convey a range of information about emotional feelings, intentions, and wishes (Horstmann 2003). Interpersonal communications are facilitated by the use of facial information as we judge others’ gender, ethnicity, attractiveness, emotions, and physical and psychological traits (Zebrowitz 2006). Facial expressions play a particularly important role in communication (Ekman 1993).

Previous studies have provided ample empirical evidence supporting the notion that smiling is associated with many positive characteristics, as compared with other facial expressions (e.g., Jones et al. 2006). For example, observers have been found to more easily remember smiling faces than faces with neutral expressions (Shimamura et al. 2006; Tsukimura and Cabeza 2008). Furthermore, individuals who smile are often evaluated as appearing younger than those who display other expressions (Hass et al. 2016; Voelkle et al. 2012). Specifically, Voelkle et al. (2012) had participants grade the age of various facial stimuli by adjusting the slider for a range of ages from 0 to 100. Hass et al. (2016) asked college student participants to categorize the emotional and neutral expressions of male facial stimuli morphed across eight age groups into categories of “young” or “old”. Their findings represent the association between smiling and youthfulness.

This association, however, might stem from a misconception. Jones and Smith (1984) argued that the eye regions convey important information. Eye regions are thought to influence age estimation because they develop wrinkles with age. Additionally, many previous studies have investigated the processes of perceiving various facial dimensions, such as expression, identity, gender, and gaze direction (Ganel and Goshen-Gottstein 2002, 2004; Ganel et al. 2005; Schweinberger and Soukup 1998), and suggest that individuals cannot consider one aspect of a face without being influenced by other aspects of the same face. George and Hole (1998) showed that changes in neighboring features, such as the eyes, nose, and mouth, influence age estimation. Therefore, Ganel (2015) hypothesized that observers actually perceive smiling faces as older than neutral faces, based on the resulting wrinkles around the eyes. If this is true, then why have smiling faces been evaluated as younger in previous studies (Hass et al. 2016; Voelkle et al. 2012)? Ganel and Goodale (2018) conducted two experiments to explain this inconsistency. In Experiment 1, participants first estimated the age of individual faces displaying only one of two potential expressions (smiling or neutral). After the perceptual estimation session (direct evaluation), participants were then asked to estimate the average age of a set of faces displaying each expression (retrospective evaluation). In Experiment 2, Ganel and Goodale added surprised faces to the facial stimuli and conducted the same procedures to replicate and extend the results of Experiment 1. Ganel and Goodale reasoned that surprised faces were perceived as younger than smiling faces in the direct evaluation because expression of surprise stretched the muscles around the eyes (Ekman et al. 1980) and decreased the number and intensity of wrinkles. In contrast, Ganel and Goodale proposed that surprised faces were perceived as older than smiling faces in the retrospective evaluation because expressions of surprise, unlike smiles, are associated with neither positive nor negative traits (Meyer et al. 1997). Through these two experiments, Ganel and Goodale found a significant interaction of smiling faces with the direct and retrospective age evaluation tasks, which influenced them in different ways. Smiling faces were perceived as older than other faces in direct evaluation, and as younger in retrospective evaluation. From these results, Ganel and Goodale claimed that the belief that smiling makes one appear younger is a misconception rooted in popular media, given that smiling faces are not perceived as younger than faces with other expressions in direct evaluation.

Ganel and Goodale’s (2018) study provided evidence that age estimation differs between direct and retrospective evaluations; however, it has not yet been verified whether the belief that smiling makes one appear younger can be generalized across cultures. Ganel and Goodale’s (2018) study had two limitations. First, Ganel and Goodale’s results may have been affected by their use of faces selected only from the Karolinska Directed Emotional Faces (KDEF) database (Lundqvist et al. 1998). In other words, their results are dependent on only one stimulus set. Second, the Israeli participants evaluated only Swedish faces, rather than faces associated with their own culture. That is, there was a cultural mismatch between the perceiver and the facial stimuli. Moreover, the rationale for Ganel and Goodale’s claims concerning the social roots of beliefs about smiling appears to be based solely on the results of one study. Therefore, we cannot determine whether this potentially biased age estimation is a universal tendency; it may be moderated by ethnicity and culture. Ganel and Goodale (2018) claimed that people believe (though mistakenly) that smiling makes faces look younger. It should be tested whether this belief is consistent across other cultures. Moreover, it is advisable to preregister replication studies so as to avoid influence by post-hoc ideas (Nosek et al. 2018). Therefore, present study is conducted as a registered replication report to confirm the findings of Ganel and Goodale in Japan and Sweden. Sweden and Japan were chosen as the nationalities of the participants and facial stimuli in our study. Sweden was used to eliminate the discrepancy between the facial stimuli and participants’ nationality that was problematic in previous studies, whereas Japan was used to test whether this belief is consistent across another culture.

Humans view facial features differently across cultures. Importantly, Western individuals tend to focus more on the eyes, whereas East Asians tend to focus more on the nose (Blais et al. 2008). Some studies showed that these tendencies vary according to the race of the target face. Western individuals made more fixations on the eye region of Caucasian faces than Asian faces, and more fixations on the nose and mouth regions of Asian faces than Caucasian faces (Goldinger et al. 2009). Similarly, Asian individuals focused more on the eye region of Caucasian faces than Asian faces, and more on the nose region of Asian faces than Caucasian faces (Fu et al. 2012; Hu et al. 2014). Thus, the eye region of Caucasian faces draws most attention in both Caucasians and Asians, whereas the nose region of Asian faces draws most attention in both Caucasians and Asians. Despite these reports on cultural differences in face perception, studies on age estimation have rarely dealt with culture, perhaps because of the limited opportunity to estimate the age of people of other cultures in real life. Therefore, we hypothesized that a new significant interaction could be observed when replicating Ganel and Goodale’s (2018) experiment with Caucasian and Asian participants and Caucasian and Asian facial stimuli. When examining the smiling faces of Caucasian individuals, Caucasian and Asian observers would pay more attention to the wrinkles that form around the eyes because of their focus on the eye region. Thus, both groups of observers would perceive the smiling faces of Caucasians as significantly older than faces with other expressions in direct evaluation. Inversely, when examining the faces of Asian individuals, Caucasian and Asian observers would attend less to the wrinkles around the eyes because of their focus on the nose region. Thus, neither group of observers would perceive the smiling faces of Asians as significantly older in direct evaluation. The present study extends Ganel and Goodale’s study to test for new interaction among the tasks, race of facial stimuli, and race of participants.

We collected data from separate groups of Japanese and Swedish participants, and in an extension of Ganel and Goodale’s (2018) study, we had subgroups from each respective population estimate the age of both Japanese and Swedish faces. The present study replicated Ganel and Goodale’s Experiment 2, in which surprised faces were added to the facial stimuli, because surprised expressions are not typically associated with either positive or negative traits (Meyer et al. 1997) and they decrease the appearance of wrinkles around the eyes (Ekman et al. 1980). Ganel and Goodale conducted Experiment 2 to replicate and extend Experiment 1 in their study. Therefore, Experiment 2 is the more comprehensive test, and the more meaningful for replication. Additionally, it is suitable to compare the effect of three facial expressions (smiling, neutral, or surprised) on age estimation to test the effect of wrinkles claimed by Ganel and Goodale. As in Ganel and Goodale’s (2018) study, we did not directly ask participants their beliefs regarding facial expressions. Rather, we asked them about the mean of the ages they responded as their estimation with respect to each expression in the retrospective evaluation task. The purpose of this procedure was to avoid inducing the social belief that smiles make people look young. Therefore, the responses in the retrospective evaluation task reflected potential participants’ beliefs regarding facial expressions.

The present study is outlined as follows. Like Ganel and Goodale (2018), we tested whether participants would rate smiling faces as older than neutral or surprised faces during direct evaluation, and whether they would rate smiling faces as younger than neutral or surprised faces during retrospective evaluation. After both age evaluation tasks, both Japanese and Swedish facial stimuli were again presented. Participants were then asked to estimate the intensity of the facial expressions one by one to test whether the intensity of facial expressions was matched across the two cultures.

We predicted two significant interactions. First, as in Ganel and Goodale’s (2018) study, we predicted a significant interaction between facial expression (smiling, neutral, or surprised) and age evaluation task (direct or retrospective). In direct evaluation, smiling faces would be evaluated as older than neutral and surprised faces because of the wrinkles around the eyes caused by smiling (Prediction 1-a). In retrospective evaluation, smiling faces would be evaluated as younger than neutral and surprised faces because of the association between smiling and youthfulness (Prediction 1-b). Second, we predicted a significant interaction among facial expression, facial stimulus (Japanese or Swedish), and age evaluation task. In direct evaluation, both Japanese and Swedish participants would significantly evaluate smiling Swedish faces as older than neutral and surprised Swedish faces (Prediction 2-a) because the eye region of Caucasian faces draws attention in both Caucasians and Asians (Fu et al. 2012; Goldinger et al. 2009; Hu et al. 2014). In contrast, participants would not significantly evaluate smiling Japanese faces as older than neutral and surprised Japanese faces (Prediction 2-b) because the nose region of Asian faces draws attention from both Caucasians and Asians (Fu et al. 2012; Goldinger et al. 2009; Hu et al. 2014).

## Method

### Ethics

The experiment was conducted in accordance with the principles of the Declaration of Helsinki (World Medical Association 2013) and the recommendations of the American Psychological Association’s Ethical Principles of Psychologists and Code of Conduct. All participants provided written informed consent before participating in the study, with the understanding that they could withdraw their participation at any time. The ethics committee of Kyushu University approved the protocol (number: 2017-003).

### Experimental design

Based on Ganel and Goodale’s research design (2018), the experiment included four independent variables: (1) “facial expressions” (smiling, neutral, and surprised); (2) “age evaluation tasks” (direct or retrospective); (3) “facial stimuli culture” (Japanese or Swedish); and (4) “participants’ nationality” (Japanese or Swedish). Facial expressions and age evaluation tasks were within-subjects variables, and facial stimuli culture and participants’ nationality were between-subjects variables. The dependent variable was facial age estimation, as rated by participants. Participants were first asked to directly evaluate the age of each type of facial expression, and then to retrospectively evaluate the average age of all the faces conveying each facial expression. For each population (Japanese and Swedish), the experiment was conducted using Japanese facial stimuli for one group of participants’ nationality and Swedish facial stimuli for the other group. The experiment was conducted in Japan and Sweden.

### Power analysis and participants

We calculated sample size based on significant interaction as measured in Experiment 2 of Ganel and Goodale’s (2018) original study. Their study showed significant interaction between expressions and tasks, and suggested that participants evaluated smiling faces as older than neutral faces in the direct evaluation condition, whereas they evaluated smiling faces as younger than neutral faces in the retrospective evaluation condition. Thus, we determined sample size based on the interaction effect size in the original study. According to Cohen (1988), η^2^ can be translated to effect size *f* by the following formula: $f = \sqrt {\frac{{\eta^{2} }}{{1 - \eta^{2} }}}$. Therefore, Cohen’s *f* = 0.29 was obtained as the effect size of Ganel and Goodale’s original raw data from Experiment 2. Using the *pwr* package in R (Champely 2012), we calculated the required sample size for interaction based on the effect size *f* = 0.29 (i.e., the effect size of the original experiment), the required alpha level α = 0.05, and the required power 1 − β = 0.95. As a result of these calculations, the required sample size for interaction was determined to be *N* = 204^1^ in total. The experiment was unable to present participants with both Japanese and Swedish facial stimuli for procedural reasons: Japanese and Swedish faces should be presented separately, but the retrospective estimation task was incidental. Thus, in each country, we collected over 51 participants to evaluate each facial stimuli culture condition (Japanese and Swedish). The total sample consisted of at least 102 Japanese and 102 Swedish participants. As a stopping rule, we set data collection not to exceed 64 for each group (i.e., a margin of the required sample size of plus 25%). Among the Japanese sample, half of the participants estimated the age of Japanese faces, and the other half estimated the age of Swedish faces. The same principle applied to the Swedish sample. Thus, we collected data from a total of four groups.

### Collected participants

In total, 240 college students in Japan and Sweden participated in this cross-cultural study based on the pre-registered experimental design. More specifically, data from 107 Japanese participants were collected, of which 54 saw Japanese faces (6 male and 48 female, *M*_age_ = 20.94 years, *SD*_age_ = 2.39, age range = 18–27 years) and 54 saw Swede faces (12 male and 41 female, *M*_age_ = 20.84 years, *SD*_age_ = 2.32, age range = 18–25 years).^2^ Data of 133 Swedish participants were collected, of which 40 saw Japanese faces (7 male, 33 female, and 27 unidentified, *M*_age_ = 24.16 years, *SD*_age_ = 3.79, age range = 19–34 years) and 40 saw Swede faces (11 male, 29 female, and 27 unidentified, *M*_age_ = 23.85 years, *SD*_age_ = 3.92, age range = 19–37 years).^3^ All participants had normal or corrected-to-normal vision. Japanese participants received \700 and Swedish participants received 75 SEK for their participation. We excluded three participants’ data from the dataset because they could not complete the experiment owing to a program error.

### Apparatus

All stimuli were presented on a computer monitor with a resolution of 1024 × 768 pixels and a refresh rate of 60 Hz. The presentation of stimuli and the collection of data were controlled by OpenSesame software operating on the computer (Mathôt et al. 2012).

### Facial stimuli

Caucasian (Swedish) facial stimuli consisted of head-and-shoulder photos of 30 women and 30 men (*M*_age_ = 25 years, ranging from 20 to 30 years) with smiling, neutral, and surprised expressions, taken from the KDEF database (Lundqvist et al. 1998). This resulted in a total of 180 photos, the same number used by Ganel and Goodale (2018). Japanese facial stimuli consisted of head-and-shoulder photos of 30 women and 30 men with smiling, neutral, and surprised expressions from the ATR Facial Expression Image Database (DB99) (ATR-Promotions, Kyoto, Japan; Ogawa and Oda 1998; *M*_age_ = 21.1 years, ranging from 20 to 24 years). Caucasian facial photos were adjusted to 7 × 9 cm and divided into three sets of 60; faces in set A were smiling, faces in set B had a neutral expression, and faces in set C had a surprised expression. Each participant was presented with 60 photos consisting of 20 individuals from each of the three sets (smiling, neutral, and surprised, respectively). Japanese facial photos were also adjusted to 7 × 9 cm and divided into three sets of 60, as in the above procedure. Individual photos were not repeated for each participant. Thus, six different counterbalancing orders were arranged (i.e., a three-factorial design) and assigned to each participant in the experiment.

### Procedure

The experiment was conducted with participants individually in a dark room. A viewing distance of 40 cm from the screen was fixed by a chin-rest. First, an instruction was presented onscreen in Japanese or Swedish, stating, “Images of various facial expressions will now be presented. Please evaluate the age of each face as accurately as possible”. A single face was presented after the participant pressed the space bar. Participants typed in their evaluation of the facial image’s apparent age via numeric keypad, and confirmed by pressing the enter key. The software used in the present study limited participants’ input to integers. Although Ganel and Goodale did not restrict responses to integers, this limitation of responses does not have a substantial effect on comparing the results with those of the original study. The input number was displayed below the image and could be modified using the backspace key. Once the enter key was pressed, another facial expression was presented. One block contained 60 images with a randomized for each participant.

Next, the instruction “Please answer the following series of questions” was presented to participants in Japanese or Swedish in the retrospective evaluation task. Participants were asked, in Japanese or Swedish, to estimate the average age of all the faces with three expressions presented to them, as follows: “As accurately as possible, please estimate and respond with the average age of all the smiling face photos you evaluated”. The same instructions were provided for the neutral and surprised facial expressions. *Participants* typed in their estimation of the average age via the numeric keypad and confirmed their response by pressing the enter key.

**Fig. 1:**
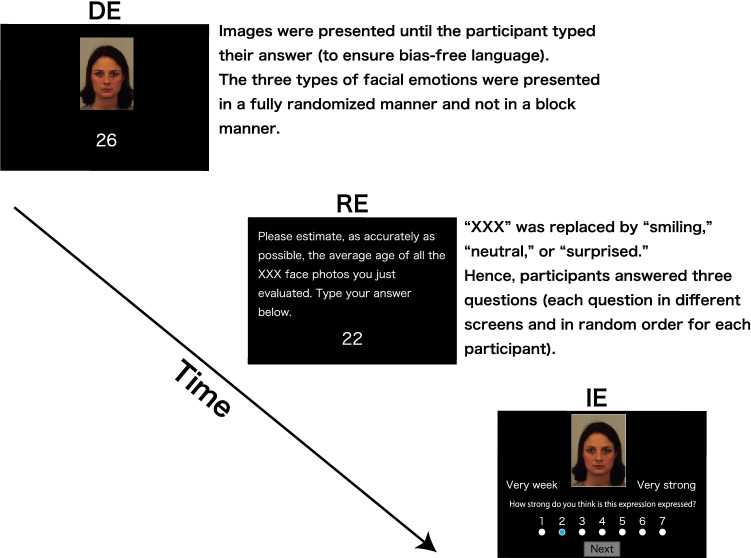
Illustration of the sequence of events in the experiment. The sequence of events was fixed to be DE → RE → IE. *DE *direct evaluation, *RE *retrospective evaluation, *IE *intensity evaluation

After both age evaluation tasks, the Japanese and Swedish facial stimuli were again presented to participants. Participants were asked to estimate the intensity of the facial expressions one by one. Next, the question “How strong do you think is this expression expressed?” and anchors in their native language were presented under the facial expressions (Fig. 1). Anchors were on a 7-point Likert scale (i.e., ranging from 1, indicating “very weak”, to 7, indicating “very strong”). The higher the score, the greater the intensity of the expression. This task tested whether the intensity of facial expressions differed across the cultures, and if so whether the differences in emotional intensity would influence the age estimation. The sequence of these procedures is illustrated in Fig. 1.

## Results

### Data analysis

Ganel and Goodale (2018) conducted a two-way repeated measures ANOVA with task (direct or retrospective evaluation) and expression (smiling, neutral, or surprised) as within-subjects factors on evaluated age value. Based on the original study, we performed a four-way mixed ANOVA with task and expression as within-subjects factors and facial stimuli culture (Japanese or Swedish) and participants’ nationality (Japanese or Swedish) as between-subjects factors. Following Ganel and Goodale’s (2018) statistical analyses, no missing value or outlier detection procedures were applied. Considering the original study’s results from Experiment 2, the present study defined the success criteria for replication as a significantly older evaluation of smiling faces than surprised faces (*p* < 0.05) in the direct condition and a significantly younger evaluation of smiling faces than surprised faces in the retrospective condition (*p* < 0.05) (see Figure 1 in Ganel and Goodale 2018). We used surprised faces as the control condition because of the ambiguity of wrinkles around the eyes in neutral faces; wrinkles were considered a determining factor for age in the previous study (Ganel and Goodale 2018), and in surprised faces, wrinkles around the eyes were visibly fewer than in the smiling faces.

Analysis that applies multiple analysis methods to a single dataset and explores only the consistent effects from those analyses is called a multiverse analysis (Steegen et al. 2016). Performing multiverse analyses can further increase the transparency of a study. We also performed the following supplementary analyses as multiverse analyses: (a) a four-way mixed ANOVA, in which missing values (if any) were eliminated with list-wise case deletion (Cheema 2014) and outliers of ± 2.5 SD from the participant’s mean in each condition were removed (see Marmolejo-Ramos et al. 2015); (b) a 2 × 3 × 2 × 2 ANOVA-type statistic on evaluated age value with task and expression as within-subjects factors and facial stimuli culture and participants’ nationality as between-subjects factors^4^; (c) a two-way repeated-measures ANOVA reanalysis of the original data obtained by Ganel and Goodale (2018) after applying missing value and outlier detection procedures (as described above)^5^; and (d) an ANOVA-type statistic reanalysis of the original data obtained by Ganel and Goodale (2018). These supplementary multiverse analyses (Steegen et al. 2016) were intended to identify patterns in the data.

## Confirmatory (pre-registered) analysis

### Data accessibility

The anonymized data set and R codes for analysis of the present study are publicly available at https://osf.io/sv9rc/.

### Data analysis

**Fig. 2:**
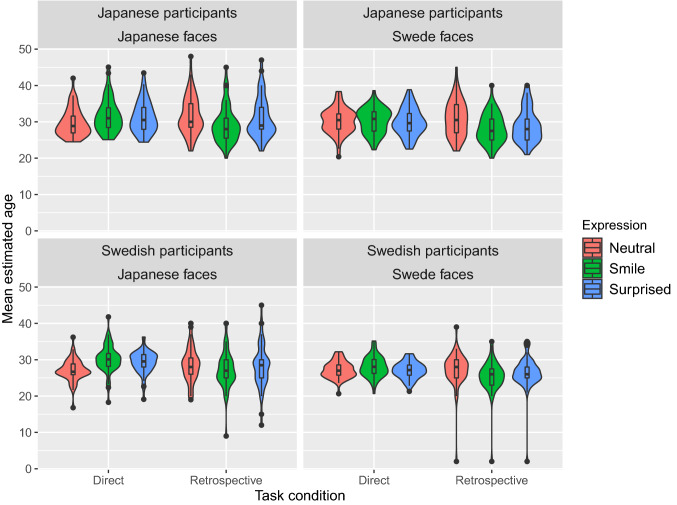
Violin and box plots showing the experimental results

Twenty-one participants’ data with missing values in the retrospective condition were also excluded from the dataset. We conducted multiple analyses following the pre-registered analysis procedures. Mean estimated ages are plotted by group in Fig. 2 as an overview of the pattern of estimated ages. As the main analysis, we conducted a four-way mixed ANOVA with task and expression as within-subjects factors and facial stimuli culture (Japanese or Swede faces) and participants’ nationality (Japanese or Swedish) as between-subjects factors. The results revealed main effects for facial stimuli culture (*F*(1, 212) = 7.39, *p* < 0.01, generalized eta squared: η_G_^2^ = 0.02), participants’ nationality (*F*(1, 212) = 30.27, *p* < 0.001, η_G_^2^ = 0.09), and task (*F*(1, 212) = 15.09, *p* < 0.001, η_G_^2^ = 0.01). We observed significant interactions between facial stimuli culture and expression (*F*(2, 414) = 8.40, *p* < 0.001, η_G_^2^ < 0.01), between participants’ nationality and expression (*F*(2, 414) = 3.76, *p* < 0.05, η_G_^2^ < 0.01), and between expression and task (*F*(2, 416) = 61.32, *p* < 0.001, η_G_^2^ = 0.03). To check whether the interaction between expression and task satisfied the pre-registered success criteria of the replication, we conducted pairwise comparisons of the expressions for the task conditions. The results showed that participants evaluated smiling faces as significantly older compared with the other facial expressions (*t*s < 6.043, *p*s < 0.01) in the direct condition (Smiling: mean = 30.07 years, *SD* = 7.66; Neutral: mean = 28.56 years, *SD* = 6.81 years; Surprised: mean = 29.38 years, *SD* = 6.81 years), whereas participants evaluated smiling faces as significantly younger compared with the other facial expressions (*t*s < 8.76, *p*s < 0.001) in the retrospective condition (Smiling: mean = 27.49 years, *SD* = 4.83 years; Neutral: mean = 29.62 years, *SD* = 5.22 years; Surprised: mean = 28.62 years, *SD* = 5.35 years). A significant three-way interaction was also found among expression, facial stimuli culture, and task (*F*(1, 424) = 4.20, *p* < 0.05, η_G_^2^ < 0.01). To verify our predictions (Predictions 2-a and 2-b), we conducted pairwise comparisons between expressions for facial stimuli culture in the direct condition. The results showed that participants significantly evaluated smiling Japanese faces as older than neutral Japanese faces in the direct condition (*t*(522) = 7.20, *p* < 0.001; Smiling: mean = 30.93 years, *SD* = 8.51 years; Neutral: mean = 28.36 years, *SD* = 7.20 years). No difference was reported between smiling and surprised Japanese faces in the direct condition (*t*(546) = 1.87, *p* = 0.10; Surprised: mean = 30.27 years, *SD* = 7.17 years). In contrast, smiling Swede faces did not differ significantly from other Swede facial expressions in the direct condition (*t*s < 2.08, *p*s > 0.06; Smiling: mean = 29.23 years, *SD* = 6.62 years; Neutral: mean = 28.75 years, *SD* = 6.40 years; Surprised: mean = 28.51 years, *SD* = 6.31 years).

In accordance with the pre-registered reports, we also conducted the following supplementary analyses.

- In accordance with the pre-registered data processing procedure, the missing values in collected data were eliminated with list-wise case deletion (Cheema 2014), and outliers of ± 2.5 SD from the participants’ mean in each condition were removed (see Marmolejo-Ramos et al. 2015).
- We performed a rank-based ANOVA-type statistical test with task and expression as within-subjects factors and facial stimuli culture (Japanese or Swede faces) and participants’ nationality (Japanese or Swedish) as between-subjects factors. The results showed a significant effect of task (*F*(1, 1187.36) = 19.15, *p* < 0.001), facial stimuli culture (*F*(1, 1187.36) = 27.39, *p* < 0.001), and participants’ nationality (*F*(1, 1187.36) = 110.65, *p* < 0.001). We also observed significant interactions between expression and task (*F*(2, 1187.36) = 25.07, *p* < 0.001), between expression and facial stimuli culture (*F*(2, 1187.36) = 5.07, *p* < 0.01), and between facial stimuli culture and participants’ nationality (*F*(1, 1187.36) = 6.16, *p* < 0.05).
- To reanalyze the original data obtained by Ganel and Goodale (2018), we conducted a two-way repeated measures ANOVA on task (direct vs. retrospective age evaluation) and expression (smiling, neutral, and surprised) after applying missing value and outlier detection procedures. The two-way ANOVA revealed the main effect of task (*F*(1, 40) = 29.77, *p* < 0.001, η_p_^2^ = 0.43) and the interaction between task and expression (*F*(1, 40) = 29.77, *p* < 0.001, η_p_^2^ = 0.42).
- We conducted a rank-based ANOVA-type statistical test with task and expression as within-subjects factors on the original data obtained by Ganel and Goodale (2018). The results revealed a significant main effect of task (*F*(1, 232.42) = 15.11, *p* < 0.001) and the interaction between task and expression (*F*(1.99, 232.42) = 11.65, *p* < 0.001).

### Face stimuli intensity

The intensity of each facial expression stimulus was also measured to test whether the intensity of facial expressions differed across cultures and whether the differences in emotional intensity would influence age estimation. To check such differences in the intensity, we conducted a three-way mixed ANOVA with expression as a within-participants factor and facial stimuli culture or participants’ nationality as between-participants factors. The results showed a significant main effect of expression (*F*(1.77, 413.33) = 660.87 *p* < 0.001, η_G_^2^ = 0.57); the intensity of neutral faces was significantly evaluated as weaker than the other facial expressions (Smiling: mean = 4.91, *SD* = 1.35; Neutral: mean = 2.63, *SD* = 1.59; Surprised: mean = 5.00, *SD* = 1.47). We also found a significant main effect of participants’ nationality (*F*(1, 233) = 24.65, *p* < 0.001, η_G_^2^ = 0.05) and interactions between participants’ nationality and expression (*F*(1.77, 413.33) = 31.92, *p* < 0.001, η_G_^2^ = 0.06). Facial stimuli culture and expression also showed a significant interaction (*F*(2, 466) = 3.99, *p* < 0.05, η_G_^2^ = 0.01); Japanese participants evaluated the intensity of surprised faces higher compared with Swede participants (*t*(594) = 2.67, *p* < 0.05; Japanese: mean = 5.06, *SD* = 1.36; Swedes: mean = 4.94, *SD* = 1.58).

## Exploratory (not pre-registered) analysis

Unexpected social conditions (COVID-19) created a new variable, the situation (Lab or Online), which was not mentioned in the pre-registered reports. Thus, we conducted additional analyses as part of a multiverse analysis to confirm the robustness of the results of the pre-registered analysis. Table 1 shows the comparisons of the result patterns of the confirmatory and exploratory analyses. We conducted linear mixed effect modelling (LME) with task, expression, facial stimuli culture, and participants’ nationality as fixed effects and participants’ ID and situation as random effects. We performed this analysis to consider the effect of the experimental situation (Lab or Online), an independent variable that was unexpectedly created. This analysis was performed using the “anova” function in R package “lmerTest.” The results showed a significant main effect of task (*F*(1, 1060) = 23.17, *p* < 0.001), facial stimuli culture (*F*(1, 212) = 7.39, *p* < 0.01), and participants’ nationality (*F*(1, 212) = 30.27, *p* < 0.001). Significant interactions were also observed between expression and task (*F*(2, 1060) = 46.98, *p* < 0.001), expression and facial stimuli culture (*F*(2, 1060) = 8.12, *p* < 0.001), and expression and facial stimuli culture (*F*(2, 1060) = 3.6324, *p* < 0.05). Moreover, we found a significant three-way interaction among expression, facial stimuli culture, and task (*F*(2, 1060) = 3.22, *p* < 0.05).

**Table 1:** Comparison of the pattern of results from analytical approaches

Variable	Analysis approaches		
	ANOVA	Rank-based ANOVA	Linear Mixed Effect (LME)
A	**	***	**
B	***	***	***
A·B			
C			
A·C	***	**	***
B·C	*		*
A·B·C			
D	***	***	***
A·D			
B·D			
A·B·D			
C·D	***	***	***
A·C·D	*		*
B·C·D			
A·B·C·D			

Cells with asterisks indicate a significant main effect and/or interaction. *A *facial stimuli culture, *B *participants’ nationality, *C *expression, *D *task, · interactions

*p < 0.05, **p < 0.01, ***p < 0.001

We performed non-parametric pairwise comparisons using the “mctp” function in the “nparcomp” R package (Noguchi et al. 2020). The comparisons for the interaction between expression and task were conducted as with the confirmatory analysis. The results revealed that participants significantly evaluated smiling faces as older compared with the neutral faces in the direct condition (*nptt* = 4.35, *ES* = 0.62, 95% CI = [0.57, 0.67], *p* < 0.001).^6^ However, smiling and surprised faces showed no difference in the direct condition (*nptt* = 1.90, *ES* = 0.45, 95% CI = [0.39, 0.50], *p* = 0.06). These results were partially consistent with our prediction (Prediction 1-a). In contrast, participants significantly evaluated smiling faces as younger than neutral (*nptt* = 4.85, *ES* = 0.36, 95% CI = [0.31, 0.42], *p* < 0.001) or surprised faces (*nptt* = 2.35, *ES* = 0.57, 95% CI = [0.51, 0.62], *p* < 0.05) in the retrospective condition. These results were entirely consistent with our prediction (Prediction 1-b). The non-parametric pairwise comparisons for the interaction among expression, facial stimuli culture, and task were also conducted as with the confirmatory analysis. The results revealed that Japanese smiling faces were evaluated as older than Japanese neutral faces in the direct condition (*nptt* = 5.05, *ES* = 0.71, 95% CI = [0.63, 0.77], *p* < 0.001). In contrast, Swede smiling faces did not differ significantly from other Swede facial expressions in the direct condition (*nptt*s < 1.66, *ES*s < 0.55, *p*s > 0.1). These results were exactly the opposite of our predictions (Prediction 2-a, 2-b).

## Discussion

In the present study, we attempted to replicate Ganel and Goodale’s (2018) study with an international comparison between Japanese and Swedish participants, in their respective countries, using face stimuli of two races (Japanese or Swede faces). First, we predicted that smiling faces would be evaluated as older than neutral and surprised faces in the direct evaluation condition because of the wrinkles around the eyes caused by smiling (Prediction 1-a), whereas in the retrospective evaluation condition, smiling faces would be evaluated as younger than neutral and surprised faces because of the association between smiling and youthfulness (Prediction 1-b). The results of our study showed support for these predictions: smiling faces were evaluated as older than neutral or surprised faces in the direct evaluation condition, but evaluated as younger than neutral or surprised faces in the retrospective evaluation condition. Second, we also predicted that both Japanese and Swedish participants would significantly evaluate smiling Swede faces as older than neutral and surprised Swede faces in the direct evaluation condition (Prediction 2-a), and in contrast, they would not significantly evaluate smiling Japanese faces as older than neutral and surprised Japanese faces (Prediction 2-b). Contrary to our prediction, the results revealed that the participants did not evaluate smiling Swede faces as older than other Swede facial expressions in the direct evaluation. Moreover, the participants significantly evaluated smiling Japanese faces as older than neutral Japanese faces in the direct evaluation. However, we found no significant difference between smiling and surprised Japanese faces in the direct evaluation. There were also no significant differences among smiling, neutral, and surprised Swede faces in the direct evaluation. In addition, the facial expressions were estimated for intensity separately to test whether their intensity matched across the two cultures. The results showed that the participants evaluated the intensity of Japanese surprised faces as higher compared with Swede surprised faces.

Our study was generally consistent with the results suggested by Ganel and Goodale (2018), although non-parametric pairwise comparisons did not find differences in facial expression per facial stimuli culture in the direct evaluation. That is, we found that smiling faces were evaluated as older than neutral and surprised faces in direct evaluation, regardless of the race of the participants or stimulus face. Meanwhile, smiling faces were evaluated as younger than neutral faces in the retrospective evaluation. These results indicated the influence of stereotypes about specific emotional expressions on evaluations of age. In our study, smiling, which was an expression of positive emotion, tended to make people remember that they looked younger. This bias was found in both Japanese and Swedish participants. Thus, the bias based on the association between “smile” and “youth” was consistent at least in the case of Japanese and Swedish participants. In addition, this erroneous belief was found despite the differences in mean age and range between the two databases used in our study. This finding suggests that similar results may be obtained using other facial stimuli. Ganel and Goodale (2018) stated that this erroneous belief can be found throughout popular culture. Indeed, Japanese advertisements reflect this mistaken belief. As such, there may be no difference in such values of beauty and appearance between modern Westernized Japan and Europe. In addition, exposure to global information media, like the internet, affects perceptions of facial attractiveness (Batres and Perrett 2014). Hence, the exposure to Western values by the mass media may have shaped the common cultural associations between “smiling” and “youth”. Given the ubiquity of mass media, only a few areas remain unexposed. The majority of the participants in our study and in Ganel and Goodale (2018) were students from universities in urbanized areas, such as Japan, Sweden, and Israel. Takahashi et al. (2017) suggested that differences in exposure to emoticons between individuals living in urban and ethnic minorities in rural areas may affect the sensitivity to emotion recognition of emoticons. Zhu et al. (2020) showed that the sensitivity of trypophobia, or the emotion of a strong aversion induced by clusters of holes or round objects (e.g., lotus seed pods), have differences between individuals living in urban and ethnic minorities in rural areas. These previous studies provide evidence that urbanization could affect emotion perception. Exposure to mass media can be considered as a part of urbanization. Future research needs to compare this bias between people from urban areas and ethnic minorities with no access to the internet.

We also hypothesized that the effect of wrinkles around the eyes on the estimation of age would vary with the stimulus race based on previous studies, which have shown that faces of different races have different attention-inducing areas (nose and eyes for Japanese and Swede faces, respectively). Thus, we predicted that Swede’s smiles would be evaluated as more aging than Japanese smiles in the direct condition. However, our results were contrary to this prediction; smiling Japanese faces were significantly evaluated as older than neutral Japanese faces in the direct condition. Meanwhile, smiling Swede faces did not differ significantly from other Swede facial expressions in the direct condition. In addition, the multiverse analysis did not consistently show significant interactions across expression, task, and facial stimuli culture. Despite the fact that different facial stimuli cultures attract attention to different regions of the face, the present study did not show differences in age estimation by facial stimuli culture. That is, our study indicated that the attention bias by the race of faces had no effect on age estimation. Alternatively, the wrinkles “around the eyes” may not have been the only factor affecting age estimation. Rather, these results may indicate an effect of the characteristics of the stimuli. Faces with greater facial contrast have been reported to look younger (Porcheron et al. 2013). Our results may be due in part to the differences in contrasts by skin color, which may have led to a significant difference between Japanese smiling and neutral faces. However, these interpretations need to be taken with caution. In the evaluation phase, the presentation time of facial stimuli was not specified. When facial expressions are presented for a short time, attentional bias may affect the estimation of age. The two countries also showed a difference in the intensity of the surprised face. However, this is not directly related to the results; it indicates potential differences in other characteristics of the facial stimuli between the two countries. In addition, the stimulus races in this study were only Japanese and Swedish, and other races were not considered. Studies reporting differences in perceptions of Asian and Western smiles have been conducted with Chinese and American nationals (Mai et al. 2011; Yuki et al. 2007). Further studies should include other races in the stimulus set.

Accurate age estimation has applications to a range of settings, from facilitating medical diagnosis to early resolution of criminal cases. Although AI algorithms for face recognition (FR) and estimation of age are being developed (e.g., Escalante-B and Wiskott 2020; Zhu et al. 2018), most of these algorithms are biased against non-Caucasian races (Cave and Dihal 2020), consequently affecting the classification of emotional facial expressions. In this regard, Rhue (2018) found that FR algorithms attribute more negative emotions to black men’s faces than white men’s faces. To our knowledge, no study has shown whether FR algorithms can estimate the age of a person accurately regardless of the person’s race (and culture; see Barrett et al. 2019) and facial emotional state. We hope our findings can inform the engineering of FR algorithms.

Constraints on the generalizability of the present findings are as follows. In the experiment, we used Japanese and Swede stimuli and participants, and cross-culturally consistent results were obtained. This practice would suggest that the generalizability of findings would be high. Nevertheless, what the study yielded was only the consistency of the results in two countries. While we recognize the importance of the visual cue of wrinkles as the cause of this age effect, facial features across countries have a very large variance, and hence, the availability of the cue varies. Further, different cultures will have different images of facial expressions. The application of our findings of this study should thus be limited to the population of the two countries in our work.

In sum, the present study was a registered replication report of a paradoxical phenomenon in which the estimated age of a face differs according to its expression, and the effect changes in opposite directions when estimated by observing the smile directly and retrospectively. Further, the present study used, as both stimuli and participants, Swede (i.e., from the face stimulus set used in the previous study) and Japanese people, who seem to have a different face processing method than Europeans. We were able to reproduce the findings of the previous study. Our findings suggest that age estimation from faces is not a unitary process that can be determined by simple factors, such as partial facial features or stereotype-based biases formed by the mass media, but by a combination of many factors. Given the robustness of this phenomenon, it is worthy of further exploration for a more detailed mechanism and to enable a higher degree of applicability.

## Data Availability

All relevant data analysed in this study are available at https://osf.io/sv9rc/

## Footnotes

1 At stage 1 of pre-registration, this required sample size was estimated based on the three-way interaction between facial expression, facial stimuli culture, and task. However, this sample size was incorrect owing to a miscalculation. The correct required sample size is *n* = 184. Fortunately, the miscalculated required sample size is greater than the correct required sample size, resulting in our study having higher power. The R code for this calculation is available at the repository https://osf.io/8asvx/.

2 The COVID-19 pandemic made it difficult to conduct the experiment in the laboratory. For this reason, we collected data from 50 Japanese participants online with the permission of the editors. The online experiments were performed and controlled using jsPsych (de Leeuw 2015), and the procedures were identical to those in the laboratory experiment.

3 We failed to obtain demographic information from 53 Swedish participants. However, we included these participant data in the analysis as well because this information was not used in the series of analyses.

4 The ANOVA-type statistic uses the ranks of the values and is robust to outliers and violation of the assumption underlying classical parametric ANOVA (Brunner et al. 2017; see also Erceg-Hurn and Mirosevich 2008). This method is implemented via the ‘rankFD’ function in the R package by the same name.

5 We thank the authors for making their data available to us.

6 In this study, “*nptt*” stands for non-parametric *t* test statistic and “*ES*” for its associated effect size.

## Code availability

The experimental program of OpenSesame software used in this study is available at https://osf.io/sv9rc/.
